# College Lodging House Supervision

**Published:** 1882-02

**Authors:** 


### COLLEGE LODGING HOUSE SUPERVISION.

The University of Oxford has lately instituted a measure which our American colleges might do well to follow, so far at least as the lesser powers of university faculties, and the relatively less important place of the student life of this country will allow. Oxford has employed a civil engineer to inspect the lodging houses tenanted by under-graduates, and has required examination of the water of their wells. While the University authorities have not the power to compel landlords to improve their houses, they are able to refuse to grant them licenses to receive students unless their recommendations are followed. We do not know that any American college has similar power of license, but the dependence which boarding house keepers in college towns feel toward the faculties, for recommendation, etc., gives, practically, to the latter much influence of this kind. The college management is in large measure responsible for the welfare of students ; and that need exists for sanitary supervision of lodgings, everyone who has had any connection with university towns knows. A case in point may be cited, where, in a university town in Central New York, typhoid clung persistently about a certain street, and finally, after much disease and some deaths, examination by a college professor showed dangerous pollution of the well water. Princeton has, we understand, since the epidemic of typhoid fever there, instituted a system of supervision of lodging houses, and our American colleges generally should consider what can be done in their several circumstances in this direction.—*Sanitary Engineer.*

